# Fitness costs and benefits vary for two facultative *Burkholderia* symbionts of the social amoeba, *Dictyostelium discoideum*

**DOI:** 10.1002/ece3.5529

**Published:** 2019-08-15

**Authors:** Justine R. Garcia, Tyler J. Larsen, David C. Queller, Joan E. Strassmann

**Affiliations:** ^1^ Department of Biology Washington University in St. Louis St. Louis MO USA; ^2^ Present address: Department of Biology New Mexico Highlands University Las Vegas NM USA

**Keywords:** Burkholderia, Dictyostelium discoideum, host–microbe interaction, symbiont fitness, symbiosis

## Abstract

Hosts and their associated microbes can enter into different relationships, which can range from mutualism, where both partners benefit, to exploitation, where one partner benefits at the expense of the other. Many host–microbe relationships have been presumed to be mutualistic, but frequently only benefits to the host, and not the microbial symbiont, have been considered. Here, we address this issue by looking at the effect of host association on the fitness of two facultative members of the *Dictyostelium discoideum* microbiome (*Burkholderia agricolaris* and *Burkholderia hayleyella*). Using two indicators of bacterial fitness, growth rate and abundance, we determined the effect of *D. discoideum* on *Burkholderia* fitness. In liquid culture, we found that *D. discoideum* amoebas lowered the growth rate of both *Burkholderia* species. In soil microcosms, we tracked the abundance of *Burkholderia* grown with and without *D. discoideum* over a month and found that *B. hayleyella* had larger populations when associating with *D. discoideum* while *B. agricolaris* was not significantly affected. Overall, we find that both *B. agricolaris* and *B. hayleyella* pay a cost to associate with *D. discoideum*, but *B. hayleyella* can also benefit under some conditions. Understanding how fitness varies in facultative symbionts will help us understand the persistence of host–symbiont relationships.

**OPEN RESEARCH BADGES:**

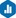

This article has earned an Open Data Badge for making publicly available the digitally‐shareable data necessary to reproduce the reported results. The data is available at https://openscholarship.wustl.edu/data/15/

## 1 INTRODUCTION

It has become increasingly clear over the past two decades that most organisms are host to a consortium of microbes, called the microbiome, and that the relationship between hosts and their microbiomes can be complex, and not always beneficial (Cani, 2018). Host–microbe interactions can run the gamut from positive to negative, with the net effect of the interaction on each partner's fitness depending on environmental context (Bronstein, 1994), the presence of other organisms (Rudgers & Strauss, 2004), and the evolutionary history of the relationship (Ishikawa et al., 2016). The positive and negative effects of microbes on hosts have been documented and studied extensively. The effect of host association on microbial fitness has not been as extensively investigated (Garcia & Gerardo, 2014; Mushegian & Ebert, 2016), although we have gained an appreciation for how host association has affected the evolution of beneficial microbial genomes (Moran, McCutcheon, & Nakabachi, 2008; Wernegreen, 2017) and metabolic pathways (Udvardi & Poole, 2013; Wilson & Duncan, 2015). Obligate symbionts often have their fitness aligned with that of their hosts [(Frank, 1997), but see (Keeling & McCutcheon, 2017)]. However, facultative symbionts, those that are acquired from the environment and can live outside the host, have independent fitness interests that often conflict with those of their host (Douglas & Smith, 1989; Garcia & Gerardo, 2014; Mushegian & Ebert, 2016). Understanding these symbiont fitness interests and accounting for the costs and benefits of symbiosis in microbes have largely been neglected. However, symbiont fitness is important for understanding how and under what conditions symbiotic host–microbe interactions persist.

There are currently multiple evolutionary explanations for the persistence of host–microbe interactions. Pathogenic infections are a common host–microbe interaction, for which hosts have evolved to resist or escape, but the interaction can persist because the microbe benefits. Long‐term persistence is more likely, though, when both the host and microbe benefit, that is, when their interaction is mutualistic. If both the host and the microbial symbiont have higher fitness when in association, then selection will act upon both partners to maintain the interaction, even though conflict persists. This has largely been assumed to operate within symbiotic host–microbe interactions in which the host benefits from the microbe. There is evidence supporting this mutualistic explanation, showing that host association is responsible for larger populations of facultative symbionts (Kuykendall, 1989; Lee & Ruby, 1994; Storelli et al., 2018). For example, living in the gut of *Drosophila* leads to a larger overall population of the bacterium *Lactobacillus plantarum* compared to just living in the environment (Storelli et al., 2018). An alternative explanation for the maintenance of host–microbe symbioses is exploitative host control, interactions in which the host benefits from a symbiont but the symbiont does not benefit [also called extortion or imprisonment (Garcia & Gerardo, 2014)]. Although mutualistic interactions commonly impose costs on symbionts, exploitative host control differs in that the costs are not offset by the benefits, when averaged across all conditions. The role of exploitative host control in stabilizing symbioses has been modeled (Frean & Abraham, 2004; Hilbe, Nowak, & Sigmund, 2013), but only a few studies have shown that it operates in natural systems (Johnson, Oldach, Delwiche, & Stoecker, 2007; Lowe, Minter, Cameron, & Brockhurst, 2016).

One reason that the effect of symbiosis on microbial symbionts has been neglected is that it can be difficult to quantify symbiont fitness and especially difficult to compare symbiont fitness in different environments (Garcia & Gerardo, 2014). Focusing on horizontal or environmentally acquired symbionts resolves some of these issues because many can be grown in culture or in nonhost environments in the laboratory (Takeshita & Kikuchi, 2017). However, it is sometimes unclear how symbiont fitness should be measured. The number or biomass of nodules on legume roots is frequently used as a rhizobial fitness proxy, but it can be problematic because the relationship between nodule size and symbiont abundance varies between rhizobia isolates (Ratcliff, Underbakke, & Denison, 2012), and nodules can contain more than one isolate (Denison & Kiers, 2011). Fungal symbionts such as arbuscular mycorrhizae and lichen fungi grow filamentously, making it hard to count individual cells (Pringle & Taylor, 2002). They can also have complex lifecycles that can include sexual and asexual reproduction. In addition, hosts can provide benefits such as dispersal (Nazir, Tazetdinova, & Elsas, 2014) or protection from predators that only have an effect on symbiont fitness in certain environments.

**Figure 1:**
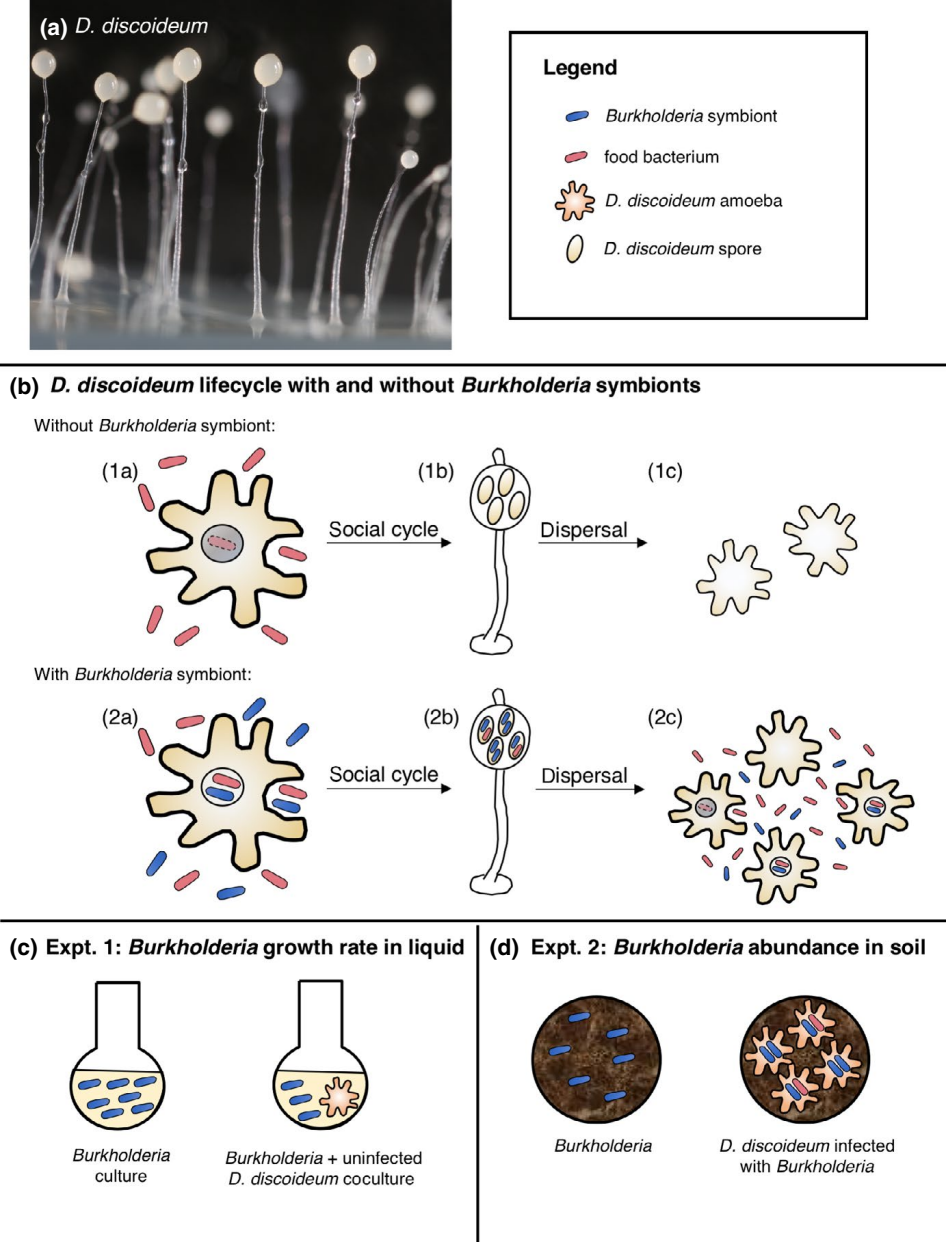
Overview of the effect of *Burkholderia* symbionts on the lifecycle of *Dictyostelium discoideum* and of the experiments done in this study. (a) *D. discoideum* fruiting bodies showing the sorus, a mass of spores and extracellular matrix, that is held aloft by the stalk. Picture taken by Tyler Larsen. (b) When *D. discoideum* is not colonized by *Burkholderia*, (1a) vegetative amoebas feed on bacteria (*Klebsiella pneumoniae* in our experiments) until they are depleted. The amoebas then aggregate to form multicellular slugs that disperse and eventually form a fruiting body for further dispersal. (1b) Spores in the fruiting body are devoid of prey bacteria. (1c) If the spores are dispersed to a location with sparse or poor quality prey, the amoebas quickly aggregate and produce few spores (Brock et al., 2011). (2a) When *D. discoideum* is colonized with *Burkholderia*, some prey bacteria remain and are carried with *Burkholderia* throughout the aggregation and dispersal of *D. discoideum*. (2b) As a result, sori are colonized by *Burkholderia* and prey bacteria. (2c) If the spores are dispersed to a location without prey, *D. discoideum* can grow and eat the descendants of the prey that were carried through dispersal. Once colonized, *D. discoideum* can carry *Burkholderia* and prey bacteria for many generations (DiSalvo et al., 2015). (c) In experiment 1, we compare the growth rates of *Burkholderia* in liquid culture alone to *Burkholderia* in liquid coculture with *D. discoideum* amoebas uninfected with *Burkholderia* (see Methods for further detail). (d) In experiment 2, we used soil microcosms to measure the abundance of *Burkholderia* added to the soil as nonsymbiotic cells or in symbiosis with *D. discoideum*. An equivalent number of *Burkholderia* were added in both treatments, and *Burkholderia* abundance was measured at four timepoints

Here, we use a simple growth‐based approach to determine the effect of host association on bacterial fitness. Although it seems intuitive, comparing bacterial abundance in host and nonhost environments (e.g., comparing symbionts in hosts and soil) can be misleading because it is difficult to determine an equivalent sampling area between the two habitats. Instead, we compare bacterial abundance in an environment with and without hosts (e.g., comparing symbionts in soil with and without hosts; Figure 1d). For the no‐host treatment, bacteria are inoculated directly into the nonhost environment and quantified after a period of growth. For the host treatment, bacteria and hosts are added to the nonhost environment and then bacteria both within and outside the host cells are quantified. This is advantageous because it accounts for effects the host has on the bacteria within its cells and in the environment. We also emphasize the use of a natural substrate for the nonhost environment instead of artificial growth media so that we can understand the host–microbe interaction in an ecologically relevant context.

The social amoeba host *Dictyostelium discoideum* and its mini‐microbiome are an ideal symbiotic system in which to quantify the effect of host association on bacterial fitness. *D. discoideum* live in soil or feces as single‐celled, vegetative amoebas. When their bacterial prey is depleted, thousands of *D. discoideum* amoebas aggregate to form a multicellular slug that disperses through the soil to form a fruiting body that holds spores aloft for further dispersal (Smith, Queller, & Strassmann, 2014; Figure 1a). In the wild, *D. discoideum* contains a small microbiome of edible and inedible bacteria (Brock, Haselkorn, et al., 2018). Of these bacteria, three *Burkholderia* species confer upon *D. discoideum* the ability to carry bacterial prey during dispersal (Figure 1b; Brock, Douglas, Queller, & Strassmann, 2011; Brock, Hubert, et al., 2018; DiSalvo et al., 2015; Haselkorn et al., 2019). This provides a fitness advantage to *D. discoideum* when there is not an acceptable food source in the new location (Figure 1b; Brock et al., 2011; DiSalvo et al., 2015), but decreases slug migration distance (Brock, Jones, Queller, & Strassmann, 2015) and is costly when a *Burkholderia* infection is newly established (Shu, Brock, et al., 2018) or when food is abundant (Brock et al., 2011). *Burkholderia* symbionts provide a further competitive advantage to their hosts by suppressing the growth of nearby *D. discoideum* uninfected with *Burkholderia* through the release of small molecules (Brock, Read, Bozhchenko, Queller, & Strassmann, 2013).

Although *D. discoideum* benefits from *Burkholderia* symbionts under certain conditions, it is unclear whether *Burkholderia* benefit or not. In *D. discoideum, –Burkholderia* are facultative symbionts that can be transmitted from host‐to‐host but can also live autonomously in nonhost environments. However, there are multiple lines of evidence that suggest *Burkholderia* symbionts could benefit from living with *D. discoideum*. *B. agricolaris* and *B. hayleyella* cells are attracted to chemicals secreted by *D. discoideum* (Shu, Zhang, Queller, & Strassmann, 2018), suggesting *it* could be a desirable host. *B. agricolaris* and *B. hayleyella* are both present within *D. discoideum* spores (Khojandi, Haselkorn, Eschbach, Naser, & DiSalvo, 2019; Shu, Brock, et al., 2018), which makes it likely that they could have an increased dispersal capability, as *D. discoideum* do (Smith et al., 2014), from sitting atop a stalk. *Burkholderia* also remain viable throughout the *D. discoideum* lifecycle and can exit from postdispersal spores and reproduce, further supporting a dispersal advantage. It is unclear what other advantages or disadvantages might apply, but potential benefits include nutrient acquisition and protection from predators or pathogens in the soil. Here, we use growth and abundance as fitness measures as a baseline determination of the costs and benefits to *B. agricolaris* and *B. hayleyella*.

In this study, we use the *D. discoideum–Burkholderia* system to investigate the effect of symbiosis on the fitness of facultative symbionts (Figure 1). We focused on two of the three *Burkholderia* symbiont species (*B. agricolaris* and *B. hayleyella*) because they encompass most of the phenotypic and genotypic diversity found in the *D. discoideum* symbionts (Haselkorn et al., 2019). In experiment 1, we measured the effect of *D. discoideum* on the growth rate of independent, nonsymbiotic *Burkholderia* cells in liquid culture (Figure 1c). Although a less realistic environment, liquid culture allowed us to take the multiple measurements necessary to accurately determine growth rate. We used *D. discoideum* that were cured of *Burkholderia* in this experiment so that established *Burkholderia* symbionts would not confound the host effect. In experiment 2, we measured the abundance of *Burkholderia* with and without *D. discoideum* hosts in soil microcosms over a time course that covers the entire *D. discoideum* life cycle (Figure 1d). In order to use a realistic host‐to‐symbiont ratio, we inoculated the host treatment with *D. discoideum* already colonized with *Burkholderia* and then added an equivalent number of independent, nonsymbiotic *Burkholderia* cells to the no‐host treatment. We found that *D. discoideum* suppressed the growth rate of all *B. agricolaris* and *B. hayleyella* isolates in liquid culture. However, *D. discoideum* had a markedly different effect on the abundance of the two *Burkholderia* species in the soil microcosms—*B. hayleyella* had larger total populations when in symbiosis with *D. discoideum*, while the total population of *B. agricolaris* was either suppressed or not affected by symbiosis. These results suggest that *B. hayleyella* is likely to be more adapted to associating with *D. discoideum*, while *B. agricolaris* may be less adapted or more likely to be taken advantage of by the host. This is unsurprising since *B. hayleyella* has a reduced genome consistent with strong host dependence. We discuss the implications these findings have for the evolution of host–microbe interactions along the mutualism–parasitism continuum and the role symbiont fitness has in maintaining symbiotic interactions.

## 2 MATERIALS AND METHODS

### 2.1 Culturing and maintenance of *D. discoideum* clones and *Burkholderia* isolates

All of the *D. discoideum* clones used in this study were collected as an effort to establish wild‐caught, non‐laboratory‐adapted *D. discoideum* clones as experimental models. They were collected from a variety of locations over a period of years, mostly by our laboratory group [but see (Francis & Eisenberg, 1993); Table 1]. Some of these clones consistently harbored *Burkholderia,* and subsequent analysis has shown that about 25% of wild *D. discoideum* harbor one or more *Burkholderia* species [though the percentage of infected *D. discoideum* varies significantly in different locations; (Haselkorn et al., 2019)]. The original, wild‐caught host and symbiont pairings have been preserved in glycerol stock, and we refer to these as “native” infections. We have found that all *D. discoideum*‐associated *Burkholderia* tested to date can infect *D. discoideum* clones other than their original host (Haselkorn et al., 2019) and we call these “non‐native” infections. In this study, all experiments were done with native host–symbiont pairings. In experiment 1, *D. discoideum* clones were cured of their native *Burkholderia* infections and re‐paired with the same *Burkholderia* isolate (see next section). All of the *D. discoideum*‐associated *Burkholderia* tested to date follow the general pattern of providing a benefit to their native *D. discoideum* when prey is not available, but imposing a cost when prey is available. However, there is significant variation in the costs and benefits provided by each *Burkholderia* isolate both in native and in non‐native hosts (Khojandi et al., 2019; Shu, Brock, et al., 2018).

**Table 1:** *Dictyostelium discoideum* clones and *Burkholderia* isolates used in this study

Clone	Native *Burkholderia* sp. (isolate)	Collection		*Burkholderia* traits				This study		References
		Location	Date	Edibility^a^	Toxicity of Cells/Supernatant^b^	In sori^c^	Food carriage^d^	Expt 1^e^	Expt 2	
QS31	*B. agricolaris* (B31)	H Arb, TX	6/6/02	Inedible	None/–f	Yes	Strong	X		Haselkorn et al. (2019)
QS159	*B. agricolaris* (B159)	MLBS, VA	5/08	Inedible	None/–	Yes	Strong; Intra‐ and Extracellular	X	X	Haselkorn et al. (2019) Khojandi et al. (2019)
QS161	*B. agricolaris* (B161)	MLBS, VA	5/08	–	–/–	Yes	Yes; Intra‐ and Extracellular	X	X	Khojandi et al. (2019)
QS175	*B. agricolaris* (B175)	H Arb, TX	7/15/04	–	–/–	Yes	Yes		X	Haselkorn et al. (2019)
QS317s	*B. agricolaris* (B317s)	H Arb, TX	6/5/01	Inedible	–/–	Yes	Yes	X		Haselkorn et al. (2019)
QS606	*B. agricolaris* (B606)	L Falls, NC	7/3/01	Inedible	Low to None/–	Yes	Strong		X	Haselkorn et al. (2019)
NC21	*B. agricolaris* (Bnc21)	L Falls, NC	7/3/01	Inedible	None/–	Yes	Strong	X	X	Haselkorn et al. (2019)
QS11	*B. hayleyella* (B11)	MLBS, VA	10/15/00	Inedible	Low to None/High	Yes	Weak	X	X	Brock et al. (2013) Haselkorn et al. (2019) Khojandi et al. (2019)
QS22	*B. hayleyella* (B22)	MLBS, VA	9/25/00	Inedible	–/Low	Yes	Yes	X		Brock et al. (2011) Brock et al. (2013) DiSalvo et al. (2015)
QS23	*B. hayleyella* (B23)	MLBS, VA	9/25/00	Inedible	–/Low	Yes	Yes	X	X	Brock et al. (2011) Brock et al. (2013)
QS155	*B. hayleyella* (B155)	MLBS, VA	5/08	Inedible	–/Low	Yes	Yes	X	X	Brock et al. (2011) Brock et al. (2013)
QS171	*B. hayleyella* (B171)	Lake I, MN	5/08	Inedible	None/–	Yes	Weak to Moderate; Extracellular	X		Brock et al. (2013) Haselkorn et al. (2019) Khojandi et al. (2019)
NC63	*B. hayleyella* (Bnc63)	LBG, NC	10/88	–	–/–	Yes	Yes		X	Brock et al. (2013) Francis and Eisenberg (1993)

Abbreviations: H Arb, Houston Arboretum; MLBS, Mountain Lake Biological Station; L Falls, Linville Falls; Lake I, Lake Itasca; LBG, Little Butts Gap.

a Inedible indicates *D. discoideum* uninfected by *Burkholderia* is unable to grow with that *Burkholderia* isolate as its sole food source.

b Toxicity of *Burkholderia* cells to *D. discoideum* uninfected by *Burkholderia* is indicated by decreased *D. discoideum* spore production when grown on 5% or 0.25% of the *Burkholderia* compared to growth only with food bacteria as reported in Haselkorn et al. (2019). Toxicity of *Burkholderia* supernatant to *D. discoideum* uncolonized by *Burkholderia* is indicated by decreased *D. discoideum* spore production when grown on a filter infiltrated with cell‐free supernatant from stationary‐phase *Burkholderia* at a concentration of OD_600_ = 1.5 compared to a filter infiltrated with starvation buffer as reported in Brock et al. (2013).

c As indicated by positive spot tests or micrographs showing fluorescently labeled *Burkholderia* in the sorus (Khojandi et al., 2019).

d Carriage of the food bacteria indicated via spot test. Strength of carriage is indicated by the number of samples with positive PCR amplification of food‐specific PCR as reported in Haselkorn et al. (2019). Intracellular or extracellular carriage is indicated by micrographs showing the location of fluorescently labeled food bacteria in the sorus as reported in Khojandi et al. (2019).

e Clones used in this assay were cured of their native *Burkholderia* infection.

f Not tested.

To get *D. discoideum* clones and *Burkholderia* isolates for our experiments, we grew them up from freezer stocks on SM/5 [2 g glucose (Fisher Scientific), 2 g Bacto Peptone (Oxoid), 2 g yeast extract (Oxoid), 0.2 g MgSO_4_ * 7H_2_O (Fisher Scientific), 1.9 g KH_2_PO_4_ (Sigma‐Aldrich), 1 g K_2_HPO_4_ (Fisher Scientific), and 15 g agar (Fisher Scientific) per liter] plates. We plated *D. discoideum* clones from glycerol stock with ~2.5 × 10^8^ cells of *Klebsiella pneumoniae* as food and grew them for ~5 days to allow all cells to complete the social cycle and form fruiting bodies. We then collected sori, the top part of the fruiting body that includes the spores (Figure 1a), into KK2 buffer [2.25 g KH_2_PO_4_ (Sigma‐Aldrich) and 0.67 g K_2_HPO_4_ (Fisher Scientific) per liter] with a sterilized loop and counted with a Neubauer hemocytometer before use in subsequent experiments. Spores were kept in KK2 buffer at 4°C for up to a month before use in experiments. We streaked *Burkholderia* isolates from glycerol stock and incubated them for two days at room temperature. Bacteria from plates were then either resuspended in KK2 buffer or used to inoculate overnight cultures for experiments.

### 2.2 Experiment 1: *Burkholderia* growth rate in liquid culture with and without *D. discoideum*

We measured the maximum specific growth rate of each *Burkholderia* isolate alone and in coculture with *D. discoideum* in a Tecan Infinite M200 PRO microplate reader (Figure 1c). The *D. discoideum* clones used had been treated to remove pre‐existing *Burkholderia*. We did this by treating the *D. discoideum* with selective antibiotics and confirming the absence of *Burkholderia* (Brock et al., 2011; DiSalvo et al., 2015). Cured *D. discoideum* clones were used in order to isolate the host's effect on *Burkholderia* and remove any effect that *Burkholderia* already within *D. discoideum* may have on the interaction. Each *Burkholderia* isolate was partnered with the cured version of its native *D. discoideum* host (i.e., the *D. discoideum* clone that hosted the *Burkholderia* isolate when they were isolated from natural soil). To prepare *D. discoideum* amoebas for the growth assay, we plated 4 × 10^4^ spores from frozen stock on SM/5 with ~2.5 × 10^8^ cells of *Klebsiella pneumoniae* as food. We harvested the amoeba cells ~36 hr after inoculation by washing the plates with KK2 buffer. We washed the amoebas three times in KK2 buffer and then incubated them in KK2 buffer with 300 μg/ml tetracycline for 1 hr shaking at 200 rpm at room temperature. This tetracycline treatment killed any extracellular *K. pneumoniae* remaining in the buffer. After a final wash in KK2 buffer, we counted the amoebas using a Neubauer hemocytometer and resuspended them at a concentration of 10^7^ amoebas/ml. To prepare the *Burkholderia* inocula, we diluted overnight cultures of each *Burkholderia* isolate to an OD_600_ of 0.1 and then added 10 μl to wells of a Nunc 96‐well tissue culture plate with 100 μl of SM/5 broth. We added 10 μl of the 10^7^ amoebas/ml suspension to the *D. discoideum* coculture treatment and 10 μl of KK2 buffer to the *Burkholderia*‐only treatment to equalize the volume. We ran three replicates of each sample. A monoculture of each *D. discoideum* clone was also included as a negative control in each experiment. Contamination of the *D. discoideum* coculture wells with residual *K. pneumoniae* is a potential problem that can be detected by including *K. pneumoniae* as a positive control. *K. pneumoniae* has a maximum specific growth rate nearly twice the average *Burkholderia* value, so we can assume that any coculture wells that had a growth curve similar to *K. pneumoniae* are contaminated and remove them; however, none of the runs included here had *K. pneumoniae*‐contaminated wells. We incubated the microplate at 22°C, and OD_600_ was measured every 15 min for 48 hr. The OD_600_ readings of amoebas grown in liquid SM/5 in monoculture never increased beyond the baseline reading, indicating only *Burkholderia* in the coculture wells contributed to the OD_600_ readings. We fitted growth curves and calculated maximum specific growth rates using fitr (https://github.com/dcangst) in R version 3.4.4 (R Core Team, 2018).

### 2.3 Experiment 2: *Burkholderia* abundance in soil microcosms with and without *D. discoideum*

We measured the abundance of *Burkholderia* with and without *D. discoideum* by inoculating sterilized soil microcosms with *D. discoideum* spores infected with their native *Burkholderia* symbiont or with *Burkholderia* alone (Figure 1d). We tested five isolates of *B. agricolaris* and four isolates of *B. hayleyella* (Table 1). We created soil microcosms by adding 200 mg of sifted, sterilized Metro‐Mix 360 soil (Sun Gro Horticulture) on top of 1 ml of starving agar [35.6 mg Na_2_HPO_4_ (Sigma‐Aldrich) and 198 mg KH_2_PO_4_ (Sigma‐Aldrich) per liter] in each well of a 24‐well plate. For the *Burkholderia* + *Dictyostelium* treatment, we inoculated each well (1.5 cm diameter) of sterilized soil with 4 × 10^4^ – 2 × 10^5^ *D. discoideum* spores. The number of spores added varied by isolate in order to add equivalent numbers of *Burkholderia* cells in both treatments. For the *Burkholderia* treatment, *Burkholderia* cells were added to each well of sterilized soil in an equivalent number to the total *Burkholderia* cells present in *D. discoideum* spores for the host treatment. This was done by quantifying the average number of *Burkholderia* cells per *D. discoideum* spore for each isolate using qPCR (see next section) and then multiplying that value by the number of spores added to each well in the *Burkholderia + Dictyostelium* treatment (average of 22 *Burkholderia* cells/spore for *B. agricolaris* and 1.2 *Burkholderia* cells/spores for *B. hayleyella*). *D. discoideum* carries the food bacterium *K. pneumoniae* when colonized by *Burkholderia* (Brock et al., 2011; DiSalvo et al., 2015), so no exogenous food bacteria were added. Three replicates of each isolate and three negative control replicates, inoculated with KK2 buffer, were also made for each timepoint. We incubated the soil microcosms at room temperature (22–23°C) under fluorescent bench lights.

We collected samples at four timepoints chosen to include different stages in the *D. discoideum* social cycle that could affect bacterial replication: (a) 24 hr postinoculation, during which *D. discoideum* spores have hatched and live in the soil as feeding vegetative amoebas, (b) 96 hr postinoculation, when most *D. discoideum* amoebas are in the process of aggregating or forming migratory slugs, but some have completed the social cycle by forming fruiting bodies, (c) 8 days postinoculation, when most *D. discoideum* have completed the social cycle and the maximum number of fruiting bodies is present, and (d) 29 days postinoculation, when *D. discoideum* fruiting bodies have started to dry and deteriorate. We removed the soil from each well, placed it in a 5‐ml tube with 2 ml of KK2 buffer, and treated each sample with 50 μM PMAxx (Biotium), a DNA‐binding dye that prevents amplification and quantification of cell‐free DNA from dead bacteria. This ensures that only live, viable *Burkholderia* can be quantified with qPCR. Samples were incubated in the dark on a platform shaker for 10 min and then incubated on ice under a 500 W light for 10 min to deactivate the PMAxx. This bulk collection of soil harvested all *Burkholderia* cells, including those that were hosted inside *D. discoideum* and those outside of *D. discoideum*. We then transferred the samples to BashingBead tubes with 0.1‐ and 0.5‐mm beads (Zymo Research), processed them in a Disruptor Genie at 3,000 rpm for 20 min, and then extracted DNA with the Quick‐DNA Fecal/Soil Microbe Kit according to the manufacturer's protocol (Zymo Research). We then determined the number of *Burkholderia* cells in each sample using qPCR.

### 2.4 qPCR assays

*Burkholderia agricolaris* and *B. hayleyella* were quantified in experiment 2 using species‐specific quantitative PCR (qPCR) assays. We designed primers specific to each *Burkholderia* species using the genomes of *B. agricolaris* isolates B317s and B1045 and *B. hayleyella* isolates B11 and B69 as a guide. We used AlleleID (PREMIER Biosoft) to design primers for genes that were identified as unique to each *Burkholderia* species using gene annotation done by JGI Integrated Microbial Genomes. The *B. agricolaris* assay amplifies a 166‐bp region of the gene for 3‐hydroxyanthranilate 3,4‐dioxygenase, an enzyme involved in tryptophan metabolism. The *B. hayleyella* assay amplifies a 150‐bp region of the *ybdD* gene, a putative DUF466 family selenoprotein. We chose these genes because they were present in the *Burkholderia* species of interest but absent in the other species, making them absolutely species‐specific. The genes were present as single copies in the genomes of *B. agricolaris* or *B. hayleyella* we have sequenced, so the assays are directly comparable. The specificity of each assay was initially determined via BLAST and was experimentally confirmed in two ways: (a) by confirming the primers did not amplify any genomic region of *D. discoideum*, *Klebsiella pneumoniae*, or the other *Burkholderia* species, and (b) by spiking samples from the target *Burkholderia* species with either *K. pneumoniae* or the other *Burkholderia* species in a concentration equal to the target sample. Assays were considered specific to one species if spiking with a nontarget sample changed the C_q_ of the target sample by 1.5% or less. We also determined the melting temperature of each assay and included melt curves in every qPCR run to ensure nonspecific products were not amplified.

We experimentally determined the optimal annealing temperature for each assay by running a dilution series on a temperature gradient. The annealing temperature that produced the best efficiency was used. We made standard curves for each assay by purifying PCR amplicons with a PureLink PCR Purification Kit (Invitrogen) and diluting them in 100 ng/μl salmon sperm DNA (Invitrogen) to make a 10‐fold dilution series spanning eight orders of magnitude. Purified amplicons were quantified with a Qubit Fluorometer and dsDNA High‐Sensitivity Assay Kit (Thermo Fisher Scientific). We tested whether the soil we used in the soil microcosm experiment had an inhibitory effect on the qPCR assays by performing spiking experiments, and determined all potential inhibitors were removed by the purification step in the Quick‐DNA Fecal/Soil Microbe Kit we used for DNA extraction.

All qPCR assays were 20 μl reactions composed of 1x iTaq Universal SYBR Green Supermix (Bio‐Rad), 300 nM each of forward and reverse primers, and 2 μl of template DNA and were run on a Bio‐Rad CFX Connect machine. The *B. agricolaris* assay consisted of primers 3haa.B1.F (5′‐CAG TGT TTG CCT CGT AAT C‐3′) and 3haa.B1.R (5′‐CTC TTC CGA CGC ATA GAA‐3′), and the *B. hayleyella* assay consisted of primers ybdD.B2.F (5′‐GTT TTC TGA TCT GCG AGA C‐3′) and ybdD.B2.R (5′‐AAT TCG TCG TAA GTC ATC AC‐3′). We ran both assays on a thermocycling program of 95°C for 3 min, followed by 40 cycles of 95°C for 10 s and 60°C for 30 s followed by a melt curve analysis from 65 to 95°C. Three technical replicates were run for each sample. No‐template controls (NTCs) were run with every assay. We analyzed all qPCR data with Bio‐Rad CFX Manager v3.1 software. The average PCR efficiency for the *B. agricolaris* runs was 90.1% and 92.1% for the *B. hayleyella* runs.

### 2.5 Statistical analyses

**Figure 2:**
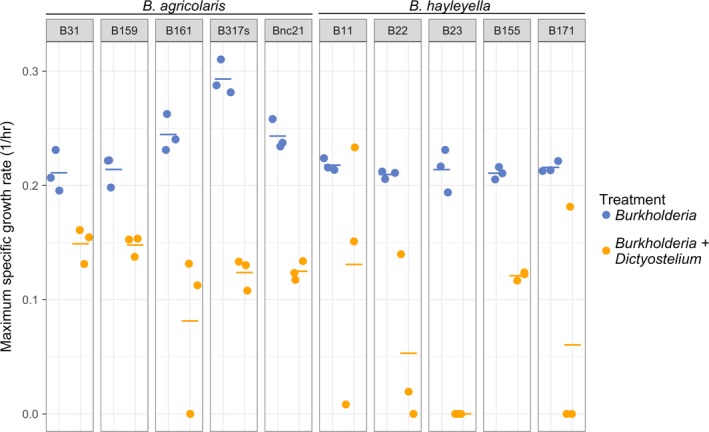
In liquid culture, *Burkholderia* have lower growth rates in coculture with *Dictyostelium discoideum* than in monoculture. Maximum specific growth rate is equal to the natural log of 2 divided by the doubling time and is determined from the maximum slope of the growth curve. Circles are replicate growth rate measurements, and lines are the median of the replicates. Points are jittered along the *x*‐axis for visibility

**Figure 3:**
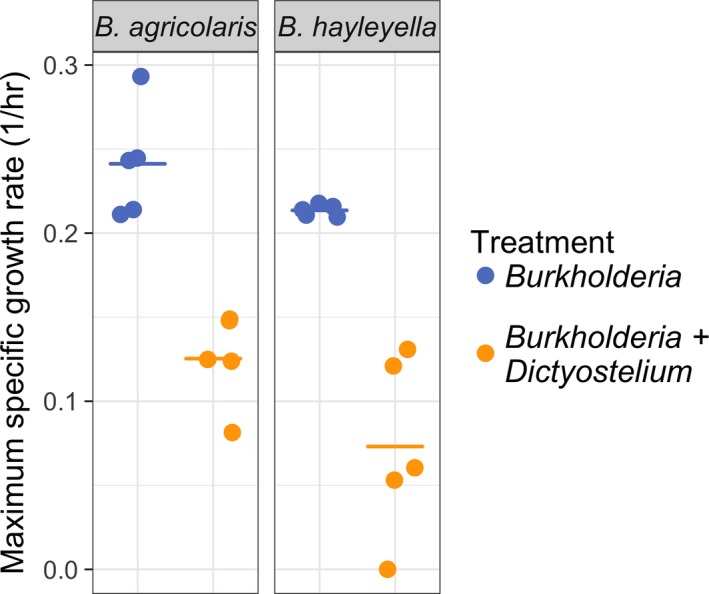
In liquid culture, *Dictyostelium discoideum* lowers the growth rate of *Burkholderia agricolaris* and *B. hayleyella. D. discoideum* significantly lowered the growth rate of *B. agricolaris* and *B. hayleyella* (Wilcoxon rank sum test, *W* = 22, *p* = 2.5 × 10^−10^), but there was no significant difference between *Burkholderia* species (Wilcoxon rank sum test, *W* = 553, *p* = 0.1294). Data are the same as in Figure 2, pooled by *Burkholderia* species. Circles are the mean maximum growth rate (*n* = 3) for each *Burkholderia* isolate, and each line is the median of all isolates within each *Burkholderia* species. Points are jittered along the *x*‐axis for visibility

**Figure 4:**
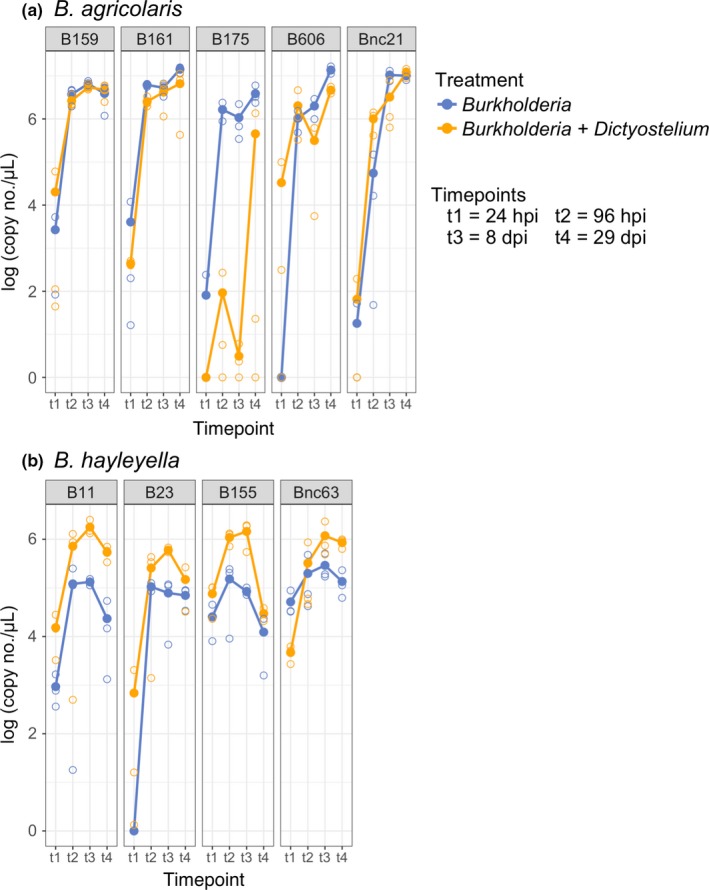
In soil, symbiosis with *Dictyostelium discoideum* significantly affects the abundance *Burkholderia hayleyella* isolates, but not *B. agricolaris* isolates. Abundance of *Burkholderia* in soil microcosms for (a) *B. agricolaris* and (b) *B. hayleyella*. We determined the abundance of each *Burkholderia* species in the entire soil microcosm, which included *Burkholderia* within *D. discoideum* and in the soil. *D. discoideum* had a significant effect on *Burkholderia* abundance (likelihood ratio test for treatment, *F* = 34.44, *p* = 8.37 × 10^−14^). The mean of replicates (*n* = 3) is indicated by large, filled circles connected with lines, and the value of each replicate is indicated by smaller, open circles. The legend applies to both (a) and (b). dpi, days postinoculation; hpi, hours postinoculation

**Figure 5:**
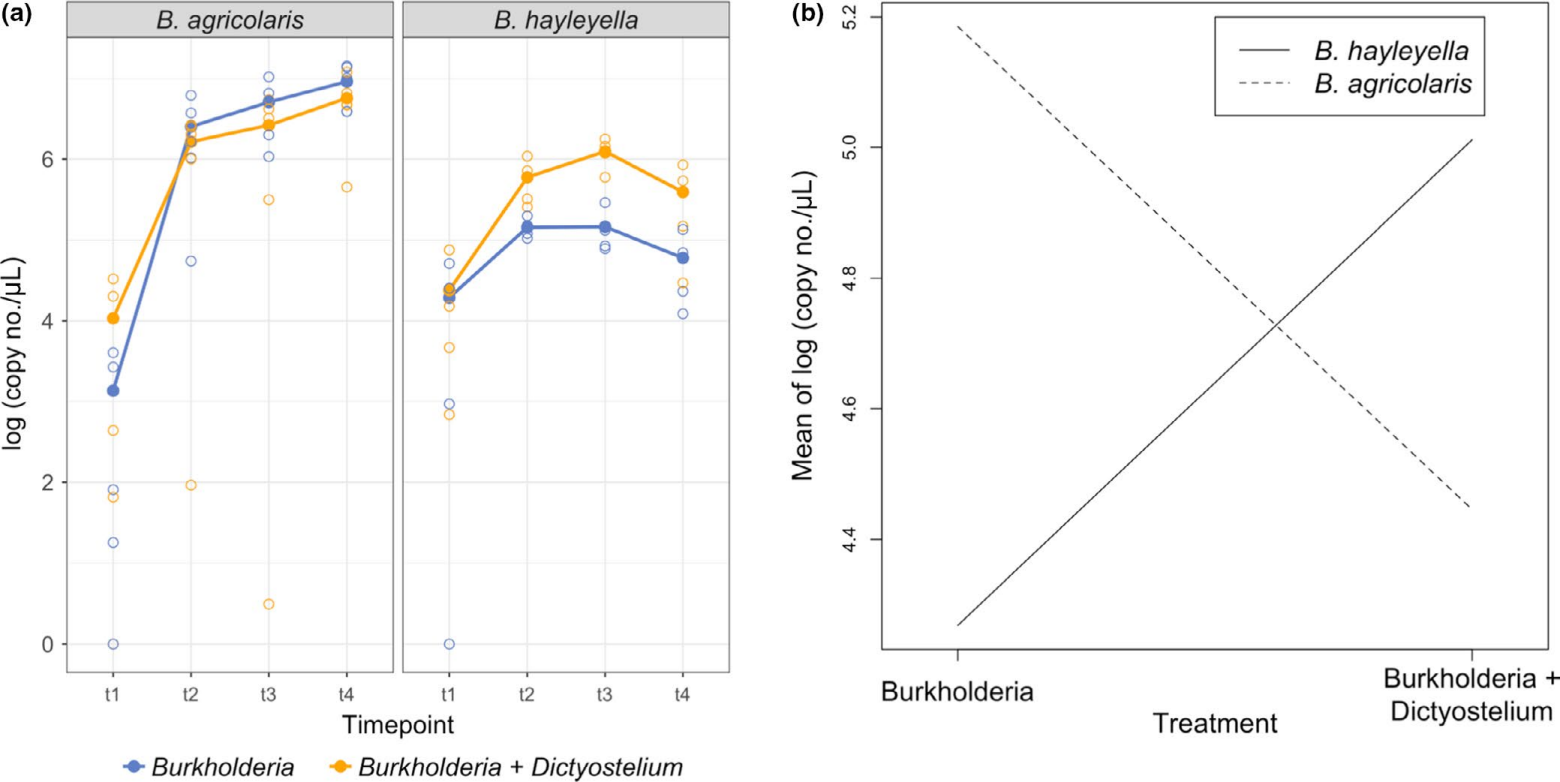
* Burkholderia hayleyella* has a larger total population size with *Dictyostelium discoideum*, while *B. agricolaris* does not. We measured the total abundance of *Burkholderia* inoculated into soil microcosms as nonsymbiotic cells (*Burkholderia* treatment) or symbiotic within *D. discoideum* (*Burkholderia* + *Dictyostelium* treatment). We determined the abundance of each *Burkholderia* species in the entire soil microcosm, which included *Burkholderia* within *D. discoideum* and in the soil. (a) Abundance of *Burkholderia* in each treatment with isolates from Figure 4 pooled by *Burkholderia* species. Filled circles are the mean *Burkholderia* abundance across all isolates, and open circles are the mean for each isolate. Timepoints are the same as in Figure 4. (b) Interaction plot for the two treatments of *B. agricolaris* and *B. hayleyella*. The endpoint of each line is the average *Burkholderia* abundance across all isolates and timepoints for the treatments in each *Burkholderia* species

The residuals for experiment 1 were not normally distributed, so we analyzed the data with a Kruskal–Wallis rank sum test with continuity correction using the kruskal.test command in R. We included both treatment and *Burkholderia* species as factors in this analysis by recoding them as a single categorical variable with four levels (*B. agricolaris* with *D. discoideum*, *B. agricolaris* without *D. discoideum*, *B. hayleyella* with *D. discoideum*, and *B. hayleyella* without *D. discoideum*; Figures 2 and 3). We then used post hoc two‐sample Wilcoxon signed‐rank tests using the wilcox.test command in R to assess the significance of treatment and *Burkholderia* species separately. *p*‐values were adjusted for multiple comparisons using the Bonferroni correction. We analyzed experiment 2 with a generalized linear model (GLM). We modeled the effect of *D. discoideum* on *Burkholderia* population size in soil microcosms (Figures 4 and 5) using the glm command in R. The model was run with a quasi‐Poisson error distribution in which the dispersion parameter was estimated to correct for overdispersion. Overdispersion was identified by comparing the sum of squared Pearson residuals to the residual degrees of freedom. Model selection was performed by determining the significance of individual and interaction terms starting with the maximal model (including timepoint, treatment, and *Burkholderia* species and all possible interactions) and then removing insignificant terms in stepwise comparisons. We determined significance of terms using likelihood ratio tests on nested models with *F* tests.

## 3 RESULTS

### 3.1 Experiment 1: *D. discoideum* suppresses the growth of both *Burkholderia* species

To test the effect of *D. discoideum* on the growth rate of *B. agricolaris* and *B. hayleyella*, we grew multiple isolates of each *Burkholderia* species in monoculture or coculture with uninfected *D. discoideum* (cured of *Burkholderia* with antibiotics). We compared the growth rates of these two treatments by determining the maximum specific growth rate (μ_max_) from growth curves. *D. discoideum* lowered μ_max_ for every isolate of *B. agricolaris* and *B. hayleyella* we tested (Figure 2), and the combination of treatment and *Burkholderia* species was significant (Kruskal–Wallis rank sum test, *χ* ^2^ = 42.7, *df* = 3, *p* = 2.8 × 10^−9^). We did two post hoc Wilcoxon tests in order to separate the effect of treatment and *Burkholderia* species. The effect of treatment was significant with *Burkholderia* growth rate suppressed when in coculture with *D. discoideum* (Wilcoxon rank sum test, *W* = 22, *p* = 2.5 × 10^−10^). *D. discoideum* seemed to lower the growth rate of *B. hayleyella* more than *B. agricolaris* (Figure 3), but this effect was not significant (Wilcoxon rank sum test, *W* = 553, *p* = 0.1294).

### 3.2 Experiment 2: *D. discoideum* supports larger populations of *B. hayleyella*, but not *B. agricolaris*

We quantified the effect of *D. discoideum* on the abundance of *B. agricolaris* and *B. hayleyella* by growing multiple isolates of each species with and without *D. discoideum* over a time course in a seminatural environment. The effect of *D. discoideum* on abundance differed by *Burkholderia* species—the abundance of *B. hayleyella* increased with *D. discoideum*, while *D. discoideum* had little effect on the abundance of *B. agricolaris* (likelihood ratio test for treatment x *Burkholderia* species, *F* = 12.93, *df* = 2, *p* = 4.96 × 10^−6^). *B. hayleyella* isolates had higher abundances with *D. discoideum* at every timepoint (Figure 4b), with one exception (Bnc63 at timepoint 1). The effect of *D. discoideum* on *B. agricolaris*, on the other hand, varied by isolate (Figure 4a). The abundances of *B. agricolaris* isolates B159 and B161 did not differ appreciably between the *Burkholderia* and *Burkholderia* + *Dictyostelium* treatments at any timepoint except the first. In two other *B. agricolaris* isolates, B606 and Bnc21, the treatment with the higher abundance switched throughout the time course and there was no clear evidence that either treatment would produce an overall larger population size. Only in isolate *B. agricolaris* B175 was it clear that the *Burkholderia* treatment consistently produced a higher abundance across the time course (Figure 4a). When all of the isolates were averaged by *Burkholderia* species, it was clear that *D. discoideum* affected *B. agricolaris* and *B. hayleyella* differently (Figure 5a). This difference was supported by a significant interaction effect between treatment and *Burkholderia* species (likelihood ratio test for treatment x *Burkholderia* species, *F* = 12.93, *df* = 2, *p* = 4.96 × 10^−6^) and is clearly evident in the interaction plot (Figure 5b). Overall, *B. hayleyella* has a higher abundance with *D. discoideum*, while *B. agricolaris* does not.

## 4 DISCUSSION

Facultative and environmentally acquired microbes are important components of many organisms' microbiomes, but it is not well understood when it is favorable for microbes to live in hosts when other habitats are available. It has been presumed that microbes benefit from host association, but the fitness of microbes in hosts and in nonhost environments has rarely been measured and compared. Here, we show that *D. discoideum* provides a fitness benefit to one facultative *Burkholderia* symbiont species by generating a larger population, while a second symbiont species does not benefit in this way. The fitness differences between these two symbiont species suggest their relationships with *D. discoideum* fall on different points of the antagonism–mutualism spectrum or reflect different levels of host adaptation.

*Dictyostelium discoideum* and the *Burkholderia* symbionts are both facultative partners, indicating they are likely to have a high potential for partner‐switching and forming new symbioses. This potential is supported by the high susceptibility of naïve or uninfected *D. discoideum* to *Burkholderia* symbionts (Haselkorn et al., 2019) and the close proximity of *Burkholderia*‐infected and *Burkholderia*‐uninfected *D. discoideum* clones in nature (Brock, Haselkorn, et al., 2018). Partner‐switching and infecting new partners require adaptations in the host or the symbiont for finding and contacting potential partners. Previous work has shown that *B. agricolaris* and *B. hayleyella* use molecules secreted by *D. discoideum* to locate and move toward the amoeba hosts for colonization (Shu, Zhang, et al., 2018).

In this study, we found that uninfected *D. discoideum* clones suppress the growth of *B. agricolaris* and *B. hayleyella* in liquid coculture. This is likely due to a secreted molecule as opposed to competition for nutrients, because wild, bactivorous clones of *D. discoideum* exhibit very little pinocytosis (ingestion of extracellular fluid) in liquid media (Bloomfield & Kay, 2016; Kessin, 2001). However, it seems counterintuitive that *Burkholderia* symbionts would be attracted to a host that can suppress their growth, but two possibilities could account for this scenario. First, the *Burkholderia* symbionts, especially *B. hayleyella* (Khojandi et al., 2019), may be largely pathogenic to *D. discoideum*, so growth inhibition may be an attempt by *D. discoideum* to resist colonization. *Burkholderia* symbionts are known to be beneficial only when *D. discoideum* have dispersed to a location without adequate or high‐quality prey, since *B. hayleyella and B. agricolaris* facilitate transport of other edible bacteria (Brock et al., 2011; DiSalvo et al., 2015; Khojandi et al., 2019). It is unknown how often this occurs, which means it is possible that *Burkholderia* symbionts are more akin to a pathogen with context‐dependent benefits.

Alternatively, growth suppression may be part of the symbiont selection and acquisition process. Many hosts employ a set of harsh, sometimes lethal, conditions that a symbiont must survive in order to colonize (Bright & Bulgheresi, 2010). For example, *Vibrio fischeri*, the bioluminescent symbiont of the Hawaiian bobtail squid, must travel through an acidic mucus matrix on the surface of the squid into ducts laden with antimicrobials, reactive oxygen species, and immune cells in order to colonize the light organ in the squid's mantle (Schwartzman & Ruby, 2016). The growth suppression of *Burkholderia* symbionts may be part of a larger mechanism used by *D. discoideum* to keep undesirable microbes out or to positively select desirable microbes. It is, however, important to note that the growth rate experiments were done in liquid culture, which is neither the ideal nor natural environment for growing wild *D. discoideum*. This environment may induce a general stress response or the secretion of molecules, such as reactive oxygen species, that are not generally reflective of *D. discoideum* interactions with *B. agricolaris* and *B. hayleyella* in the soil.

Hosts, due to their larger size and lower potential to evolve in response to symbionts, have generally been predicted to be the partner exerting control over the formation and maintenance of mutualisms (Douglas, 2010; Sachs, Mueller, Wilcox, & Bull, 2004), especially in environmentally acquired mutualisms (Kiers, Rousseau, West, & Denison, 2003). For example, some hosts produce symbiont‐controlling antimicrobials in their symbiont organs (Login et al., 2011; Mergaert, Kikuchi, Shigenobu, & Nowack, 2017; Park et al., 2018; Wang, Wu, Yang, & Aksoy, 2009), impose sanctions on or expel underperforming symbionts (Baghdasarian & Muscatine, 2000; Kiers et al., 2003; Sachs et al., 2010), or direct symbiont development toward metabolite production at the cost of symbiont reproduction (Kereszt, Mergaert, & Kondorosi, 2011). The differences we found in the ability of *B. agricolaris* and *B. hayleyella* to grow with *D. discoideum* may actually be differences in the ability of *D. discoideum* to control *Burkholderia* symbiont titers. The larger population of *B. hayleyella* in the presence of *D. discoideum* (Figures 4b and 5) may be due to higher titers within the host cell or from *B. hayleyella* exiting host cells. *D. discoideum* clones cured and re‐infected with *B. hayleyella* have a higher percentage of colonized spores (nearly 100%) than clones cured and re‐infected with *B. agricolaris*, and also have *Burkholderia* in the extracellular matrix of the sorus (Khojandi et al., 2019; Shu, Brock, et al., 2018). Additionally, as sori age, the number of spores decreases and the number of food bacteria increases in *D. discoideum* infected with *B. hayleyella*, but not *B. agricolaris* (Khojandi et al., 2019), indicating bacterial replication or other factors may lead to spore breakdown and symbiont escape. Since we did a bulk measurement of *Burkholderia* in the entire soil microcosm for this experiment, we do not know what percentage of the *Burkholderia* were in the host and what percentage were in the soil. However, the general propensity for spores to break down and for bacteria to exist and replicate extracellularly in *D. discoideum* infected with *B. hayleyella* (Khojandi et al., 2019; Shu, Brock, et al., 2018) suggests a fraction of *B. hayleyella* cells may be able to leave the host to live in the soil. Understanding whether *B. hayleyella* benefits from *D. discoideum* by remaining in the host or by taking host resources and moving into the soil is key to understanding whether the *B. hayleyella*–*D. discoideum* relationship is antagonistic or mutualistic.

In this study, we found costs and benefits to symbiont growth, but *B. agricolaris* and *B. hayleyella* could benefit from *D. discoideum* in other ways, such as an increased dispersal capability. *D. discoideum* forms fruiting bodies at the end of its social lifecycle to aid in its own dispersal (Smith et al., 2014). Both *B. agricolaris* and *B. hayleyella* are present in the spores (Khojandi et al., 2019; Shu, Brock, et al., 2018) and are viable after dispersal (Brock et al., 2011; DiSalvo et al., 2015), and so are likely viably dispersed with *D. discoideum*. *D. discoideum* spores can potentially be moved over a range of kilometers by animal vectors, which is likely much further than *Burkholderia* symbiont cells can move on their own. This potential benefit should be further evaluated in the future as it is likely a factor in the co‐occurrence and interaction between *D. discoideum* and *Burkholderia* symbionts.

Taken together, our results indicate that associating with *D. discoideum* is costly to *B. agricolaris* growth, while *D. discoideum* has both costs and benefits to *B. hayleyella* growth. It has been hypothesized that host–microbe interactions evolve toward more benefit [at least for the host; (Ishikawa et al., 2016)] and more fitness alignment between the partners, but that does not always seem to be the case (Chong & Moran, 2016; Keeling & McCutcheon, 2017; Minter et al., 2018). The relationship between *D. discoideum* and the two *Burkholderia* symbiont species then seems to be evidence of ongoing power struggles between partners. While *B. hayleyella* benefits more than *B. agricolaris* from *D. discoideum*, the opposite is true from the host perspective—*D. discoideum* benefits more from *B. agricolaris* than from *B. hayleyella* (Brock et al., 2011; DiSalvo et al., 2015; Khojandi et al., 2019). It is unclear whether this fitness misalignment/conflict is due to recency of establishment. Phylogenetic placement suggests *B. hayleyella* has been evolving in isolation from related *Burkholderia* for a long time (Brock, Hubert, et al., 2018; Haselkorn et al., 2019), perhaps as a result of being an intracellular symbiont of *D. discoideum*. The genome sizes of the *Burkholderia* symbionts also align with this possibility. *B. hayleyella* genomes (JGI/IMG Genome IDs 2703719273 and 2703719279) are half the size of the genomes of *B. agricolaris* (JGI/IMG Genome IDs 2703719271, 2703719272, 2703719274 – 78, 2703719280, 2703719281, 2703719283) and many other *Burkholderia* species (Mannaa, Park, & Seo, 2018), suggesting the possibility that *B. hayleyella* has evolved a reduced genome as many other intracellular symbionts, including the fungal endosymbiont *Burkholderia rhizoxinica* (Mannaa et al., 2018), and pathogens have (McCutcheon & Moran, 2011; Moran, 2002). However, the relationship between evolutionary history and the fitness consequences of interactions needs to be further investigated in this symbiosis and in general.

Some facultative symbionts, such as rhizobia in terminally differentiated root nodules (Mergaert et al., 2006) and algal cells subjected to kleptoplasty (Pierce & Curtis, 2012), seem to suffer unequivocally from associating with certain hosts. Most facultative symbionts, however, are more complicated. There is plenty of evidence that hosts transfer nutrients to symbionts (Ceh et al., 2013; Denison & Kiers, 2011; Graf & Ruby, 1998; Yellowlees, Rees, & Leggat, 2008), but few studies have examined whether these benefits are parlayed into higher fitness via larger total populations. Of those that have, there has been evidence both for and against increased symbiont fitness from host association. Associating with *Drosophila* leads to a larger total population (environmental and host‐associated populations combined) for the facultative gut symbiont, *Lactobacillus plantarum*. However, the “environmental” substrate tested in that study was yeast media, which may limit the conclusion's applicability to natural populations (Storelli et al., 2018). In one of the most convincing studies of increased symbiont fitness, release of rhizobia from senescing soybean nodules led to populations of rhizobia in the soil five times larger than in fields where plants were removed or in fields that did not include plants (Kuykendall, 1989). Overall, associating with bobtail squid hosts leads to an increase in *Vibrio fischeri* symbionts within ocean environments that contain squid (Jones, Maruyama, Ouverney, & Nishiguchi, 2007; Lee & Ruby, 1994), but further investigation has indicated there is strain‐level differentiation that has led to a trade‐off between growth in the squid host and growth in seawater (Wollenberg & Ruby, 2012). These data suggest that free‐living and symbiotic bacteria may actually be two specialized subpopulations of one bacterial species, but it is not clear how widespread this phenomenon is in facultative symbionts (Denison & Kiers, 2004). Finally, there is convincing evidence for exploitative host control of symbionts in *Paramecium bursaria* where *Chlorella* sp. symbionts have lower abundances and lower photosynthetic efficiency in hosts compared to free‐living counterparts across a range of ecological conditions (Lowe et al., 2016). Overall, there may not be a general rule for host effects on the fitness of facultative symbionts and each interaction will need to be evaluated on a case‐by‐case basis.

Here, we present evidence that hosts can provide fitness benefits to facultative symbionts. This is consistent with the “reciprocated benefits” hypothesis of interspecies mutualism, where each partner pays a cost to interact with the other, but overall the interaction results in a net benefit to the symbionts. However, we found a growth benefit accrued only to one symbiont species, while the second symbiont species only had costs to growth from host association. In addition, we used growth rate and abundance as fitness measures in this study, but there are other aspects of the interaction, such as the likely increased capacity for dispersal, that could shed more light on this interaction and remain to be tested. These different interactions may be the result of varying degrees of host adaptation, but further work is necessary to fully understand the evolutionary history between *D. discoideum* and *Burkholderia* symbionts.

## CONFLICT OF INTEREST

The authors declare no competing financial interests.

## AUTHOR CONTRIBUTIONS

JRG, TJL, DCQ, and JES conceived of and designed the experiments. JRG and TJL performed the experiments. JRG analyzed the data and wrote the first draft of the manuscript. JRG, TJL, DCQ, and JES revised the manuscript and approved the final version.

## Data Availability

All data are available through the Washington University in St. Louis Digital Research Materials Repository (https://openscholarship.wustl.edu/data/15/).
